# Detection of Infectious Hepatitis E Virus in Retail Pork Pâté After Optimization of Extraction Protocols

**DOI:** 10.3390/v18070760

**Published:** 2026-07-11

**Authors:** Tatjana Locus, Michael Peeters, Steven Van Gucht, Thomas Vanwolleghem, Ellen Lambrecht

**Affiliations:** 1 Fisheries and Food, Technology and Food Unit, Flemish Research Institute for Agriculture (ILVO), Brusselsesteenweg 370, 9090 Melle, Belgium; 2 Sciensano, Infectious Diseases in Humans, Viral Diseases, Engelandstraat 642, 1180 Ukkel, Belgiumsteven.vangucht@sciensano.be (S.V.G.); 3 Viral Hepatitis Research Group, Laboratory of Experimental Medicine and Pediatrics, University of Antwerp, Drie Eikenstraat 655, 2650 Edegem, Belgium

**Keywords:** hepatitis E virus, soft extraction method, infectious virus, foodborne pathogen, retail pork liver pâté

## Abstract

Hepatitis E virus (HEV) is an emerging foodborne pathogen in Europe, primarily associated with the consumption of pork products. While conventional destructive TRIzol extraction methods followed by RT-qPCR can detect HEV RNA in processed meat, this approach fails to distinguish between infectious and inactivated virus, complicating risk assessment. Currently, no standardized method exists for extracting intact, infectious HEV from pork products, limiting our ability to evaluate viral infectivity post-processing. In this study, we developed and evaluated three extraction methods to recover intact, infectious HEV from pork meat products. Our optimized soft extraction method using TGBE buffer and stomacher-based homogenization allowed us to successfully isolate infectious HEV from pork liver pâté bought in a Belgian supermarket, confirming the presence of infectious HEV in a ready-to-eat pâté intended for direct consumer consumption. Furthermore, viral RNA was detected following extraction with this method at concentrations (copies/g) similar to those obtained with the TRIzol method, while preserving viral integrity, as confirmed by capsid integrity assays and cell culture infectivity models. As a critical tool for assessing the impact of food processing on viral infectivity, the method holds significant potential for advancing food safety research and improving risk evaluations related to HEV transmission through pork products.

## 1. Introduction

Hepatitis E virus (HEV) represents a substantial global health concern, with over 20,000 acute clinical cases documented in Europe over the past decade [1,2]. The majority of European infections are attributed to locally acquired HEV genotype 3 (gt3) [1,3]. While many infections remain asymptomatic, HEV can lead to severe acute hepatitis and chronic disease, particularly in immunocompromised individuals such as solid organ transplant recipients [4]. Zoonotic transmission of HEV gt3 is primarily associated with pork consumption, reflecting the virus’s prevalence in swine populations [5]. Beyond domestic pigs, HEV gt3 is characterized by a notably broad host range, having been detected in numerous other animal species, including wild boars and other game animals [5]. As a result, the consumption of game meat has also been recognized as a potential route of HEV transmission to humans, alongside contact with pigs or consumption of pork products.

Some methods have been proposed for the extraction of HEV from meat products, yet there is currently no standardized extraction protocol [6,7]. Existing methodologies are predominantly designed for HEV RNA extraction to facilitate molecular detection via RT-(q)PCR [6,7]. These methods, while effective in removing inhibitory substances from food matrices for downstream molecular processing, often involve harsh chemical treatments that destroy HEV particles to release the RNA, thus preventing their use in subsequent infectivity assays [6,8,9]. However, there has been a recent shift toward gentler extraction approaches that move beyond RNA-focused methods and show encouraging potential for recovering intact HEV from pork meat samples [10,11].

Developing an extraction method that preserves HEV integrity is essential, as destructive extraction methods for RNA detection via conventional RT-qPCR give no information on virus infectivity in the sample. Implementing such a “soft” (i.e., non-destructive) extraction method, in conjunction with direct (e.g., in vitro assays) and/or indirect (e.g., capsid and genome integrity assays) infectivity detection techniques [12], can improve the relevance of results by better assessing the presence of intact, infectious HEV particles in food samples.

Extraction of intact HEV particles from pork liver has been done by others for use in various cell culture models [13,14,15,16] and an in vivo pig model [17]. Meat products (such as liver pâté and sausage), however, are challenging complex matrices that include fats, salts, and other components, which need to be removed to prevent inhibition of downstream assays, e.g., cell cultures. Although some researchers previously succeeded in isolating and culturing HEV from pig liver sausages [14,18], these cases remain limited, and a routine extraction protocol applicable to processed pork products has not yet been established.

The goal of this project was to optimize a “soft” extraction protocol for pork meat products by testing pâté, salami and dried ham without damaging the viral particles themselves. Such a method would enable direct and/or indirect infectivity assays to quantify the remaining infectious HEV in food samples. Removing inhibitory components from samples is crucial, as substances like fat are detrimental to cell cultures and can inhibit molecular detection processes [6,19]. Finally, we evaluated the soft extraction method on a naturally contaminated pork liver pâté purchased at a Belgian supermarket, to illustrate its suitability for detecting infectious HEV at retail level.

## 2. Materials and Methods

### 2.1. Viral Extraction Protocols

To evaluate the efficacy of the extraction protocols, an HEV-RNA-positive pork liver pâté from a Belgian supermarket (identified during a previous monitoring study [20]) was evaluated for remaining infectious HEV.

**Figure 1:**
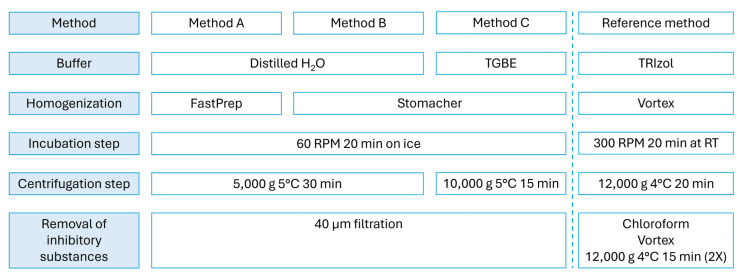
The “soft” viral extraction methods tested to extract intact HEV from pork liver pâté samples (method A, B and C) and the destructive TRIzol reference method (right).

Three extraction parameters—buffer type, homogenization method, and centrifugation step—were tested (Figure 1). Selection of these parameters was based on available virus extraction protocols from the literature [21,22]. The pork liver pâté was cut into small pieces, and fat was manually removed using a scalpel. Samples were divided into 3 g and homogenized in either 15 mL distilled H_2_O (Methods A, B) or TGBE (Tris glycine 1% beef-extract, pH 9.5 ± 0.2) buffer.

Homogenization was performed with a FastPrep^®^-24 tissue homogenizer (Method A, MP Biochemicals, Irvine, CA, USA) or a Stomacher apparatus (Methods B, C, BagMixer by Interscience, Saint-Nom-la-Bretèche, France). For homogenization with the FastPrep^®^-24, samples were placed in 50 mL tubes containing Lysing Matrix M zirconium oxide ceramic spheres (MP Biochemicals) and processed for 20 s at 6 m/s. For Stomacher homogenization, samples were placed in 400 mL filter bags and homogenized at speed 4 for 1 min.

Viral elution was performed by incubation for 20 min with constant shaking (60 RPM) on ice (Methods A, B, C).

After elution, samples were transferred to 50 mL falcon tubes and centrifuged at 5000 g at 5 °C for 30 min (Methods A, B) or 10,000 g at 5 °C for 15 min (Method C). Supernatants were collected and filtered through a 40 µm filter (Greiner, #542040, Vilvoorde, Belgium).

Viral RNA concentrations (copies/g) obtained with the soft extraction methods (Methods A, B, and C) were compared with those obtained using the conventional, destructive TRIzol-based reference method. For this purpose, virus extraction by the TRIzol method was conducted as previously described [20]. In brief, 2 g of naturally contaminated HEV pork liver pâté was mixed with 7 mL TRIzol (Invitrogen, Merelbeke, Belgium), vortexed, incubated, and centrifuged (4 °C, 12,000 g for 20 min) to pellet food particles. Supernatants were then treated with chloroform, mixed thoroughly, and centrifuged again (4 °C, 12,000 g for 15 min, repeated two times) to remove residual fat. Finally, the upper phase was collected.

All samples were stored at 5 °C for short-term or −80 °C for long-term storage and used for cell culture experiments and HEV RNA quantification by RT-qPCR (see further). Experiments were conducted in duplicate, with PBS (Phosphate-Buffered Saline, Thermo Fisher, #10010023, Merelbeke, Belgium) serving as a negative control per extraction method.

### 2.2. Assessment of HEV Capsid Integrity Assay

To evaluate the integrity of the HEV capsid, viral extracts obtained from the naturally contaminated pork liver pâté using method C were pretreated with Platinum (IV) chloride (PtCl_4_) as previously described [12]. PtCl_4_ (Sigma Aldrich, #379840, Hoeilaart, Belgium) was prepared as a 50 mM stock solution in dimethyl sulfoxide (DMSO), aliquoted, and stored at −20 °C until use. For pretreatment, 50 µL of PtCl_4_ was added to 200 µL of each virus sample to achieve a final concentration of 10 mM. The samples were then incubated on ice for 30 min with continuous shaking at 300 RPM. Following incubation, viral RNA was extracted and analyzed as detailed in subsequent sections. RNase-free water was included as a negative control in all experiments.

### 2.3. Pâté, Salami and Dried Ham: Sensitivity of Extraction Method C

To determine the sensitivity of the optimal extraction protocol (Method C), three types of pork meat products were tested: pork liver pâté, salami, and dried ham. These were purchased from Belgian supermarkets in 2023 and confirmed to be HEV-negative by RT-qPCR. These products were artificially spiked with HEV at concentrations of 10^6^, 10^5^, 10^4^, and 10^3^ genome copies per gram and subsequently processed using Method C. Experiments were conducted in triplicate, with PBS serving as a negative control.

Virus stocks used for spiking were derived from supernatants collected at seven-day intervals from A549 cells persistently infected with the HEV genotype 3 strain 47832c. The A549 cell line and HEV strain were kindly provided by Prof. Dr. Reimar Johne (German Federal Institute for Risk Assessment, Germany) [23,24]. Cells were cultured in maintenance medium: Minimal Essential Medium (MEM, Thermo Fisher, #31095-029), supplemented with 5% fetal bovine serum (FBS, Sigma-Aldrich, #F7524), 1 mM sodium pyruvate (Sigma-Aldrich, #S8636), 100 U/mL penicillin (Thermo Fisher, #15140122), and 100 µg/mL streptomycin (Thermo Fisher, #15140122). Cultures were maintained at 37 °C in a 5% CO_2_ atmosphere. Collected supernatants were stored at −80 °C until further use.

### 2.4. Cell Culture: Viral Infectivity

The presence of infectious HEV in extracts obtained using Method C was evaluated using a previously optimized cell culture assay [12]. For this purpose, extracts from spiked pork liver pâté, salami, and dried ham (10^6^ copies/gram), as well as extracts from the naturally contaminated pork liver pâté, were included in this analysis.

Briefly, A549-D3 cells were seeded in 96-well plates and cultured for 3 days in maintenance medium (as described above) at 37 °C and 5% CO_2_. Cells were then inoculated with 100 µL of virus extract or medium (negative control) in duplicate. After 1 h, cells were washed and maintained in infection medium (maintenance medium supplemented with 2% dimethyl sulfoxide (DMSO) and 5% FBS).

After five days, supernatants were collected, and cells were fixed and stained for HEV ORF2 capsid protein (Merck-Millipore, MAB8002, Hoeilaart, Belgium). Fluorescence images were processed and analyzed qualitatively using ImageJ (v1.54f) [25].

### 2.5. RNA Extraction and Detection

Viral RNA was extracted using the QIAamp Viral RNA Mini Kit (Qiagen, #52906, Germantown, MD, USA) for cell culture samples and the RNeasy Mini Kit (Qiagen, #74106) for pork liver pâté, salami and dried ham samples, according to the manufacturer’s instructions.

HEV RNA was detected via a one-step RT-qPCR protocol, as previously described [12]. Each 20 µL reaction consisted of 5 µL of extracted RNA and 15 µL of MasterMix, prepared with TaqMan^TM^ Fast Virus 1-Step Master Mix (Thermo Fisher, #4444432) and the following primers and probe: forward primer HEV-AB-F (5′-CGG TGG TTT CTG GGG TGA-3′, 0.5 µM), reverse primer HEV-AB-R (5′-GCR AAG GGR TTG GTT GG-3′, 0.5 µM), and probe HEV-pr-MGB (5′-FAM-ATT CTC AGC CCT TCG C-MGB-3′, 0.25 µM) [26]. Thermal cycling conditions included reverse transcription at 50 °C for 5 min and initial denaturation at 95 °C for 3 min, followed by 45 cycles of 15 s at 95 °C and 30 s at 60 °C.

For quantification, a standard curve was generated in each RT-qPCR run using a 10-fold serial dilution series (10^7^ to 10 genome copies per reaction) of a synthetic gBlock (IDT) containing a 1019 bp artificial HEV gt3 DNA fragment corresponding to a region of ORF2. The limit of detection (LOD) was established at 1.8 log genome copies/mL, determined by testing serial dilutions of target RNA and identifying the lowest concentration detected in ≥95% of replicates. Assuming 100% extraction efficiency across all steps, this corresponds to an estimated theoretical minimum detectable concentration of approximately 2 log genome copies/g.

## 3. Results and Discussion

The objective of this study was to develop a “soft” extraction method capable of isolating intact HEV particles from pork meat products, thereby allowing for assessment of viral infectivity through molecular (e.g., capsid integrity) and infectivity (e.g., cell culture) analyses.

As proof of principle, we assessed the performance of the developed soft extraction methods (A, B, and C) by isolating and culturing hepatitis E virus from a naturally contaminated ready-to-eat pork liver pâté collected from Belgian supermarkets [20], thereby demonstrating the presence of infectious HEV in a ready-to-eat pork product.

**Figure 2:**
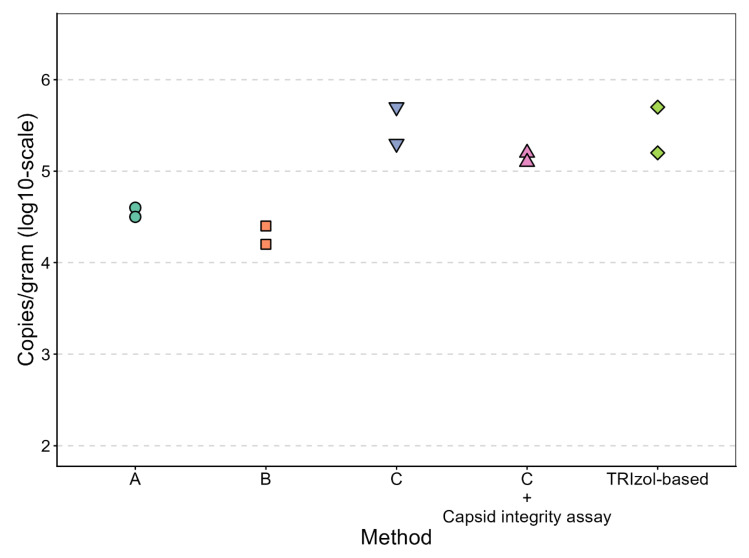
Comparison of HEV viral RNA concentrations obtained from a naturally contaminated pork liver pâté using soft extraction methods A, B, and C versus the destructive TRIzol-based extraction method. Method C was further combined with a capsid integrity assay to confirm the release of intact viral particles (n = 2).

The viral RNA concentrations (copies/gram) obtained with the soft extraction methods were compared to the destructive TRIzol-based reference method. Method C yielded viral RNA concentrations in the same range as the destructive method (5.50 [5.67–5.34] and 5.44 [5.70–5.17] log copies/gram, respectively (average [range])), while Methods A and B yielded lower concentrations (4.53 [4.59–4.46] and 4.29 [4.38–4.19] log copies/gram, respectively) (Figure 2).

Method A, which employs the FastPrep technique, applies greater force to homogenize the sample compared to the Stomacher machine, which may have contributed to partial degradation of viral particles and RNA, resulting in lower viral RNA concentrations. However, since Method A also used a different buffer than Method C, this comparison is difficult to make, and the individual contribution of the homogenization method cannot be confirmed.

Methods A and B utilize distilled H_2_O as the solution buffer, whereas Method C employs TGBE as its buffer. TGBE was selected based on its use in the ISO 15216 protocol for extracting HAV and Norovirus from soft fruits [22]. Previous studies have demonstrated that TGBE outperforms other buffers for virus extraction from food products and surfaces [27,28]. Research suggests that an alkaline buffer with a pH between 9 and 10.5 can enhance viral particle detachment from various food matrices [27,29], while the inclusion of beef extract has also been associated with improved extraction efficiency [30]; these factors could offer a possible explanation to the trend toward improved performance observed with the TGBE buffer relative to distilled H_2_O in this study.

Subsequently, extracts obtained using Method C—selected as the best-performing “soft” extraction protocol—were analyzed for capsid integrity. The results revealed that a significant portion of the detected RNA originated from viral particles with intact capsids (Figure 2). Specifically, viral RNA concentrations of 5.15 [5.24–5.07] log copies/g were detected following the capsid integrity assay, compared to 5.50 [5.67–5.34] log copies/g detected using Method C without this additional treatment.

These results suggest that Method C is indeed “gentle” on the virus, causing little to no capsid damage and preserving viral integrity. Integrity-assessment methods provide more meaningful results to estimate residual virus infectivity than conventional RT-qPCR, which does not differentiate between intact, potentially infectious virus particles and inactivated virus [12]. Our results align with other recent approaches aiming to preserve HEV integrity during extraction from pork products. A recently developed solvent-free extraction method for HEV from pig liver achieved recovery rates of up to 82% using a surrogate virus, but validated solely by RT-qPCR without assessing viral infectivity [10]. Our Method C, by contrast, was validated for infectivity preservation through both capsid integrity assays and cell culture replication, and was applied to a more complex matrix (pork liver pâté) than raw pig liver.

Similarly, another recent study used a gentle FastPrep-based extraction to recover infectious HEV from artificially contaminated sausages for cell culture titration during inactivation studies [11]. While their approach shares our goal of preserving infectivity, it was applied only to spiked samples, whereas our method was additionally validated on a naturally contaminated retail product, supporting its applicability for real-world surveillance.

A limitation of our study is that the destructive TRIzol reference method was not evaluated in combination with the capsid integrity assay. However, given that TRIzol’s phenol–guanidinium formulation is known to fully inactivate viruses and disrupt proteinaceous structures, testing this would most likely not have added useful information [31]. In addition, although viral recovery was normalized to copies per gram of starting material, we acknowledge that the use of different sample masses in the extraction protocols (i.e., 3 g in the soft extraction methods versus 2 g in the destructive TRIzol reference method) may have influenced extraction efficiency through matrix effects, inhibitor loads, or extraction kinetics, potentially affecting the comparability of the methods.

Finally, to evaluate the sensitivity of Method C, HEV-negative pork liver pâté, salami, and dried ham were spiked with an HEV sample initially adjusted to 10^6^ HEV RNA genome copies per gram. This sample was subsequently diluted 10, 100, and 1000-fold, yielding concentrations of 10^5^, 10^4^, and 10^3^ HEV RNA genome copies per gram; these dilutions were then used to inoculate the HEV-negative pork products.

The lowest HEV spiking concentration detectable by RT-qPCR in all three replicates was 10^4^ copies/g for pork liver pâté and 10^3^ copies/g for both salami and dried ham.

**Figure 3:**
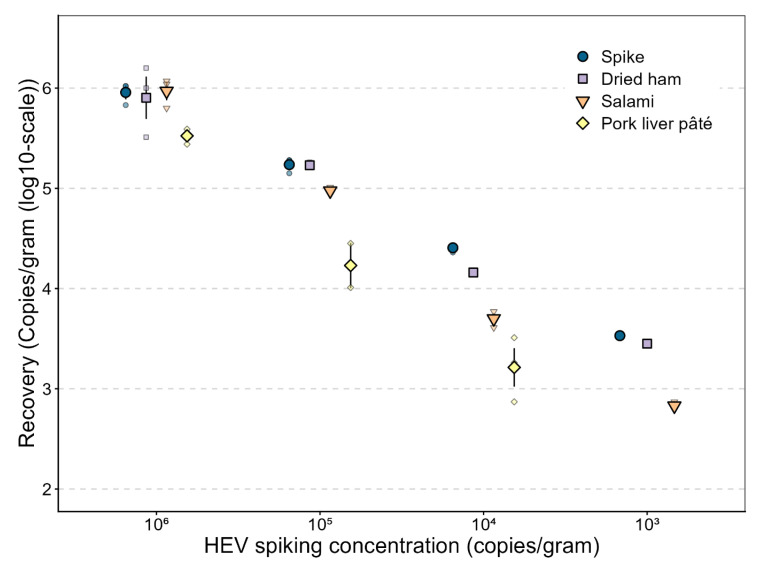
Sensitivity assessment of extraction method C on spiked pork meat products. Products were spiked with an HEV sample of 10^6^ genome copies per gram and 10-, 100-, and 1000-fold dilutions of this original sample. Quantification was performed by RT-qPCR. Means with standard error are shown, with smaller points representing individual data points (n = 3).

For pork liver pâté, mean extraction recovery at the highest spiking concentration (10^6^ copies/g) was 36% (range: 30–42%), decreasing to 11% (range: 6–16%) at the 10-fold dilution and 8% (range: 7–13%) at the 100-fold dilution; HEV RNA was no longer detected at the 1000-fold dilution in any replicate (Figure 3).

For salami, mean recovery was 104% (range: 68–126%) at the highest spiking concentration, 54% (range: 50–58%) at the 10-fold dilution, 20% (range: 16–23%) at the 100-fold dilution, and 13% (range: 0–22%) at the 1000-fold dilution (Figure 3).

For dried ham, mean recovery was 104% (range: 35–170%) at the highest spiking concentration, 98% (range: 94–104%) at the 10-fold dilution, 57% (range: 54–60%) at the 100-fold dilution, and 61% (range: 13–87%) at the 1000-fold dilution.

In this study, HEV-spiked samples were used as an internal reference for assessing extraction reproducibility. Previous in-house, unpublished experiments employing the Mengovirus extraction control (Mengo Extraction Control, ceeramTools) yielded highly variable and predominantly very low recovery rates when spiked into the naturally contaminated pork liver pâté prior to extraction (n = 2 per method; Method A: 0.02% and 5.37%; Method B: 0.01% and 0.21%; Method C: 0.25% and 0.09%; TRIzol reference method: 12.29% and 8.86%). In contrast, HEV-spiked samples showed reproducible detection across replicates, indicating consistent performance within our extraction workflow for this matrix. We acknowledge, however, that HEV itself does not constitute a conventional exogenous process control, as it is not independent of the analyte under investigation. This approach is therefore methodologically distinct from the use of a true process control virus, and for the naturally contaminated sample specifically, no independent process control was available to verify extraction efficiency.

While the present study compared complete extraction protocols rather than individual parameters, future work systematically varying a single variable at a time (e.g., homogenization method/duration, buffer and centrifugation) could help further optimize Method C and clarify the relative contribution of each step to extraction efficiency.

Additionally, increasing the number of replicates per condition would allow for statistical analysis and further strengthen confidence in the trends observed here.

To achieve one of the primary goals of the “soft” extraction method, assessing residual HEV infectivity in cell culture, pork meat extracts were inoculated onto A549-D3 cells.

**Figure 4:**
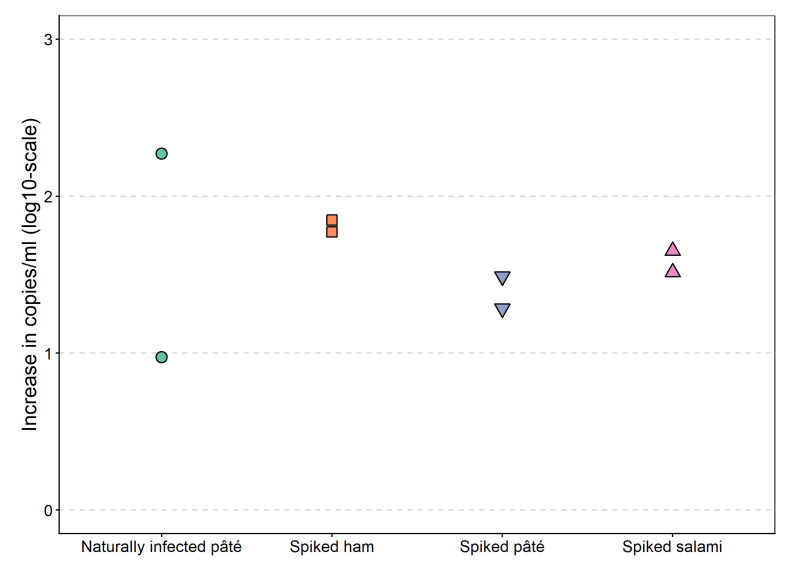
Increase in HEV RNA concentrations in cell culture supernatant five days post-inoculation. Increase in HEV RNA concentrations (log_10_ copies/mL) observed between day 0 and day 5 post-infection in A549-D3 cells inoculated with extracts from spiked dried ham, spiked salami, spiked pork liver pâté, and naturally contaminated pork liver pâté. Individual data points are shown (n = 2).

**Figure 5:**
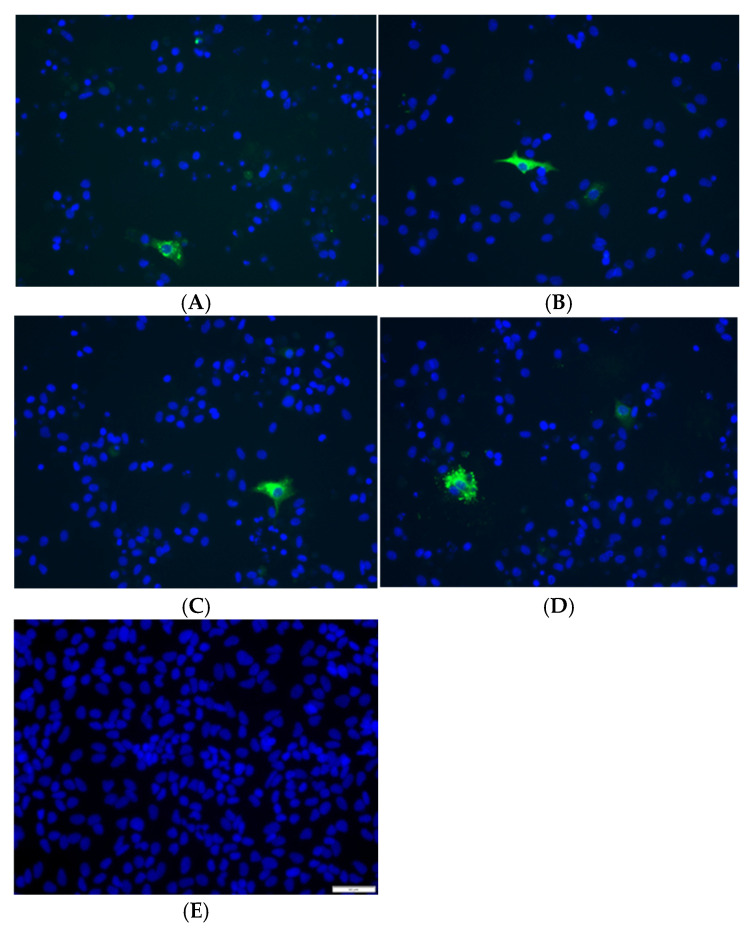
Immunostaining results for A549-D3 cells inoculated with extracts from (**A**) spiked dried ham, (**B**) spiked salami, (**C**) spiked pork liver pâté, (**D**) naturally contaminated pork liver pâté, and (**E**) HEV-negative cell culture medium (negative control). Nuclei stained with DAPI (blue) and HEV ORF2 capsid protein staining (green). The scale bar is 50 μm.

Extracts of the spiked (highest HEV concentration) pork liver pâté, salami, and dried ham were ½ diluted to minimize matrix-derived inhibitors and tested using the cell culture model. RT-qPCR results of the cell supernatant showed average increases of 1.8 [1.77–1.85], 1.6 [1.51–1.65], and 1.4 [1.28–1.49] log copies/mL for spiked dried ham, salami, and pork liver pâté, respectively, after 5 days (Figure 4). Additionally, immunostaining confirmed the presence of infected cells, indicating that the extraction protocol is indeed gentle and successfully retrieves intact viral particles that remain infectious and capable of growth in cell culture (Figure 5A–C).

Finally, extracts from the naturally contaminated pork liver pâté were evaluated using the cell culture assay. Infected cells were visualized using immunostaining, confirming the presence of infectious virus (Figure 5D). Additionally, an average increase of 1.6 [0.97–2.27] log copies/mL was observed in cell culture supernatants 5 days post-inoculation, indicating viral reproduction (Figure 4). This finding is particularly significant, as it demonstrates that ready-to-eat supermarket pork liver pâté could still contain infectious HEV. A comparable outcome was reported by Hakze van der Honing and colleagues, who identified infectious HEV in a naturally contaminated pork sausage, although their study employed a different cell culture system [14]. These results highlight the potential risk of HEV infection associated with the consumption of ready-to-eat pork products. However, it should be noted that infectious HEV was confirmed in only a single pork liver pâté sample in this study. As such, these findings should be regarded as a proof-of-concept demonstration of infectious HEV detection in a retail product, rather than an estimate of its prevalence among such products.

Although the majority of HEV infections are asymptomatic, HEV-related complications—including acute hepatitis, extrahepatic manifestations, and chronic infection—are more likely in vulnerable populations, underlining the need for targeted awareness, diagnostic efforts, and prevention strategies in risk groups.

In addition to host-related factors, viral determinants also contribute to disease severity. Notably, disease severity has recently also been linked to viral clade in a nationwide study. Here it was found that HEV clade efg infections outweigh the previously identified host risk factors of age and gender for disease severity, compared to clade abchijklm [4]. This emphasizes the importance of considering both host and viral characteristics when assessing risk and designing public health interventions.

Additionally, caution is warranted, because the minimal infectious dose in humans remains undetermined and likely varies with host factors, such as immune status, and viral factors, including genotype and strain. Individuals with compromised immune systems may be more susceptible to HEV infection, with lower infectious doses—compared to those required for healthy individuals—potentially leading to clinical symptoms [32].

Our study found that the ready-to-eat pork liver pâté purchased from a Belgian supermarket contained approximately 5 log_10_ HEV RNA copies per gram, and was able to replicate in cell culture, suggesting that retail products may (at least in this case) harbor a substantial HEV RNA load associated with recoverable infectious virus.

Although the minimal infectious dose of HEV genotype 3 in humans is not known, the concentration detected in the pâté (~5 log_10_ copies/g) represents a biologically plausible infectious load. Experimental data in pigs, HEV’s natural reservoir, indicate that inocula in the range of 10^5^–10^6^ genome copies are sufficient to establish infection [33]. Moreover, other foodborne non-enveloped enteric viruses with similar environmental stability, such as norovirus and hepatitis A virus, possess infectious doses in the order of 10–100 particles [34]. Combined with well-documented outbreaks caused by consumption of contaminated pork liver products, these comparative observations strongly support the likelihood that 5 log_10_ copies/g constitutes a realistic infectious dose for HEV in a foodborne context.

Additionally, strain variability may influence infection outcomes, as seen in norovirus, where only a few strains are responsible for most infections [35]. Notably, HEV subtypes differ in clinical impact, with clade efg associated with more severe disease presentations than clade abchijklm [4].

Despite the risk of foodborne transmission, currently, there is no official ISO protocol for extracting HEV from food products, although such protocols exist for HAV and norovirus [22]. An official HEV extraction protocol is under consideration, but this protocol is focused solely on providing food extracts for downstream conventional RT-qPCR detection methods and does not address the extraction of intact virus particles from pork meat samples. So, in practice, HEV detection in food products relies exclusively on RT-qPCR, a technique that does not necessarily correlate with the amount of infectious virus in a sample. This is even more the case in processed meat products, where a significant portion of detected viral RNA likely originates from inactivated viruses.

Effective release of HEV particles from the meat matrix is crucial, especially for processed meat products, where HEV can be found distributed within the product. Notably, a key limitation of spiking experiments is that process control viruses are typically applied onto the surface of the sample or added to homogenates shortly before extraction. As a result, these viruses may not undergo the same interactions with the matrix as viruses that are naturally present and distributed within the product during production. Naturally contaminated samples may contain viruses embedded within fat, protein networks, or tissue structures, potentially leading to stronger virus–matrix interactions and reduced extractability. An important strength of this study is the inclusion of both artificially spiked and naturally contaminated pork products. Demonstrating successful HEV detection across these matrices confirms that our extraction protocol performs reliably not only under controlled experimental conditions but also when applied to naturally contaminated samples, thereby supporting its suitability for real-world surveillance and food safety applications.

To conclude, we successfully developed such a “soft” extraction method for isolating intact HEV particles from pork meat products, facilitating the assessment of residual viral infectivity through subsequent molecular and cell culture analyses. The results demonstrated the effectiveness in preserving viral integrity while minimizing the presence of inhibitory food components. Additionally, the detection of infectious HEV in ready-to-eat pork liver pâté in retail underscores the potential risk of HEV transmission through the consumption of contaminated pork products. Our research group already demonstrated that current meat processing techniques might not be sufficient to completely destroy HEV in pork meat products such as pork liver pâté and dried sausages [36]. Therefore, further comprehensive studies are needed to fully understand the dynamics of HEV inactivation in pork liver pâté.

Overall, the developed method will advance research on the presence of infectious HEV in pork meat products and contribute to the ongoing initiatives aimed at improving the safety of pork meat products.

## Data Availability

The original contributions presented in this study are included in the article. Further inquiries can be directed to the corresponding author.
